# Relevance of Leptin and Other Adipokines in Obesity-Associated Cardiovascular Risk

**DOI:** 10.3390/nu11112664

**Published:** 2019-11-05

**Authors:** Manuel F. Landecho, Carlota Tuero, Víctor Valentí, Idoia Bilbao, Magdalena de la Higuera, Gema Frühbeck

**Affiliations:** 1 Department of Internal Medicine, General Health Check-up Unit, Clínica Universidad de Navarra, Avenida Pío XII, 36, 31008 Pamplona, Navarra, Spain; mflandecho@unav.es (M.F.L.); ibilbaodel@unav.es (I.B.); 2 Department of Surgery, Bariatric and Metabolic Surgery Unit, Clínica Universidad de Navarra, 31008 Pamplona, Navarra, Spain; ctuero@unav.es (C.T.); vvalenti@unav.es (V.V.); 3 Instituto de Salud Carlos III, CIBER Fisiopatología de la Obesidad y Nutrición (CIBEROBN), 31008 Pamplona, Navarra, Spain; 4 Obesity and Adipobiology Group, Instituto de Investigación Sanitaria de Navarra (IdiSNA), 31008 Pamplona, Navarra, Spain; 5 Department of Endocrinology and Nutrition, Clínica Universidad de Navarra, 28027 Madrid, Spain; mhiguera@unav.es; 6 Metabolic Research Laboratory, Department of Endocrinology & Nutrition, Clínica Universidad de Navarra, 31008 Pamplona, Spain; 7 CIBER Fisiopatología de la Obesidad y Nutrición (CIBEROBN), Instituto de Salud Carlos III, 28029 Pamplona, Spain

**Keywords:** obesity, adipokines, leptin, resistin, visfatin, osteopontin, adiponectin, omentin-1, ghrelin, obestatin

## Abstract

Obesity, which is a worldwide epidemic, confers increased risk for multiple serious conditions including type 2 diabetes, nonalcoholic fatty liver disease, and cardiovascular diseases. Adipose tissue is considered one of the largest endocrine organs in the body as well as an active tissue for cellular reactions and metabolic homeostasis rather than an inert tissue only for energy storage. The functional pleiotropism of adipose tissue relies on its ability to synthesize and release a large number of hormones, cytokines, extracellular matrix proteins, and growth and vasoactive factors, which are collectively called adipokines known to influence a variety of physiological and pathophysiological processes. In the obese state, excessive visceral fat accumulation causes adipose tissue dysfunctionality that strongly contributes to the onset of obesity-related comorbidities. The mechanisms underlying adipose tissue dysfunction include adipocyte hypertrophy and hyperplasia, increased inflammation, impaired extracellular matrix remodeling, and fibrosis together with an altered secretion of adipokines. This review describes the relevance of specific adipokines in the obesity-associated cardiovascular disease.

## 1. Introduction

The World Health Organization (WHO) has defined obesity as an abnormal or excessive fat accumulation that presents a risk to health and describes it as one of today’s most blatantly visible—yet most neglected—public health problems, which has reached epidemic proportions [1,2]. Obesity is a major public-health challenge. It is wide spread particularly in urban settings, and has clinical implications with potential negative effects on almost every organ system, as well as being a psychosocial and economic burden [3,4,5,6]. Furthermore, the diagnosis and management of obesity are often difficult or unsuccessful because obesity has a multifactorial nature [7]. Obesity results from a combination of genetic, epigenetic, physiological, behavioral, sociocultural, and environmental factors that lead to an imbalance between energy intake and expenditure [8]. Since 1995 at the 27th Bethesda Conference, obesity is classified as a category II risk factor, which means that the proper management of obesity is likely to lower the incidence of cardiovascular disease (CVD) events. Consistently, the American Heart Association has identified obesity as an independent cardiovascular risk factor [9]. 

Despite its definition and relevance, the body mass index (BMI) is the most common strategy to evaluate the degree of obesity, but is not always completely precise in the body composition assessment. BMI only estimates obesity based on a person’s weight (in kilograms) divided by the square of his or her height (in meters) regardless of his or her muscle mass. Furthermore, population-based studies have demonstrated a significant increased risk of CVD, independent of other traditional risk factors, for BMI-based obese patients [10]. However, people who are in the same BMI category can have quite diverse levels of health and risk factors [11]. Accumulating evidence suggests that, although the risk of all-cause mortality and cardiovascular events might be higher in people with metabolically healthy obesity compared with metabolically healthy people of a normal weight, the risk is substantially lower than in individuals with metabolically unhealthy obesity [12]. This fact strengthens the idea that obesity is a complex disease, which requires a new multi-dimensional approach to integrate and understand all of the underlying mechanisms that cause obesity [7]. Therefore, every person with obesity should be motivated to achieve a normal weight in the long term, but more moderate weight loss sufficient for the transition from metabolically unhealthy obesity to metabolically healthy obesity might also lower the risk of adverse outcomes [12].

The observed differences between subjects with the same BMI are mainly due to inter-individual variability in adipose tissue composition, distribution, and physiology [13]. Adipocytes comprise 35–70% of adipose mass, and the other cell types found in the stroma-vascular fraction include preadipocytes, mesenchymal stem cells, macrophages and other immune cells, endothelial cells, and smooth muscle cells [14]. Composition and distribution of fat mass are closely related to its physiology [15]. 

## 2. Adipose Tissue Physiology

The distribution-based classification of adipose tissue has identified two main categories known as visceral and subcutaneous adipose tissue, which can both be further subdivided. Visceral adiposity refers to an excess of intra-abdominal adipose tissue accumulation, and is part of a phenotype including expanding dysfunctional subcutaneous adipose tissue and ectopic triglyceride storage closely related to clustering cardiometabolic risk factors [16]. In general, the visceral adipose secretome has higher levels of pro-inflammatory factors, and lower levels of anti-inflammatory mediators than the subcutaneous adipose-derived secretome. Age, gender, genetics, and ethnicity are broad etiological factors contributing to the variation of visceral adipose tissue accumulation, but other specific mechanisms are responsible for proportionally increased visceral fat storage when facing positive energy, sex-hormones, local cortisol production in abdominal adipose tissues, endocannabinoids, growth hormone, and more [16]. The importance of this classification is highlighted by studies revealing different physiologies depending on the specific adipose tissue depots, and epidemiological studies that link visceral adipose tissue expansion and cardiometabolic risk. Visceral obesity, as determined by an increased waist-to-hip ratio, is associated with an increase in cardiovascular risk [17].

Moreover, the lipolytic characteristics of the diverse adipokines regardless of the different depots, in particular the perivascular and the epicardial fat, needs to be considered in view of the impact on cardiovascular disease (CVD) [18,19,20]. The adipose tissue secretome is largely depot-specific and the secretome can also be affected by systemic and local factors linked to inflammation, insulin resistance, obesity, and more [18]. Furthermore, within visceral fat, epicardial adipose tissue exhibits specific transcriptomic signatures in the periventricular, peri-atrial, and peri-coronary sites [21]. Adipose tissue expansion in obesity entails some biological and morphological changes, hyperplasia, and hypertrophy, which can ultimately lead to adipose tissue dysfunction. Adipose tissue hypertrophy, i.e., increase in cell size, and hyperplasia, i.e., increase in the cell number contributes to adipose tissue enlargement and are associated with the development of metabolic derangements and elevated cardiovascular risk in obesity [20,22,23]. Increased adipocyte volume in obese patients due to positive energy balance, such as overfeeding or a sedentary lifestyle [24], is associated with an impaired mitochondrial function and changes in membrane proteins as well as higher cell death and inflammation. The pathological expansion of adipose tissue in obesity is associated with increased infiltration of activated macrophages, neutrophils, foam cells, proinflammatory Th1 and Th17 CD4, B cells, mastocytes, and dendritic cells [14]. Other hallmarks of adipose tissue inflammation in obesity are increased adipocyte cell death, due to apoptosis and autophagy, and adipose tissue fibrosis. These morphologic changes entail specific adipokine patterns that are closely related with the metabolic effects of obesity. Additionally, other substances such as myokines and lipokines, which are beyond the scope of this review, may also be relevant when discussing the endocrine function of adipose tissue. The term “lipokines” refers to lipid species that interact with fatty acid binding proteins and lipid chaperones that dictate the partitioning of lipids inside cells, as well as play critical roles in systemic metabolism. Consistently, circulating trans-palmitoleate is associated with lower insulin resistance, the presence of atherogenic dyslipidemia, and incident diabetes [25,26,27]. Adaptive changes of skeletal muscle in response to physical activity include adjustments in the production and secretion of muscle-derived bioactive factors, known as myokines. These myokines not only act locally in the muscle in an autocrine/paracrine manner, but are also released to the bloodstream as endocrine factors to regulate physiological processes in other tissues. Myokines as irisin, myostatin, IL-4, IL-6, IL-7, and IL-15, myonectin, follistatin-like 1, or a leukemia inhibitory factor have emerged as major determinants of insulin sensitivity, mediators of fat-browning, and regulators of thermogenesis and energy expenditure. Irisin, which is derived from the cleavage of the FNDC5 protein, constitutes a myokine that induces myogenesis and fat browning (switch of white adipocytes to brown fat-like cells) together with a concomitant increase in energy expenditure. Besides being a target for irisin actions, the adipose tissue also constitutes a production site of FNDC5. Interestingly, irisin secretion from subcutaneous and visceral fat depots is decreased by long-term exercise training and fasting, which suggests a discordant regulation of FNDC5/irisin in skeletal muscle and adipose tissue. Accordingly, our group has recently reported that the adipokine leptin differentially regulates FNDC5/irisin expression in skeletal muscle and fat, which confirms the crosstalk between both tissues. Moreover, irisin secretion and function are regulated by other myokines, such as follistatin or myostatin, as well as by other adipokines, including fibroblast growth factor 21 and leptin [28].

The adipose tissue is now recognized as a crucial regulator of cardiovascular health, mediated by the secretion of several bioactive products, including adipokines, with a wide range of effects on the cardiovascular system, which can become dysregulated in obesity [18]. Adipose tissue products from the diverse fat depots are released into the bloodstream and can reach distant sites (e.g., heart and arteries), where they exert their biological effects in an endocrine manner. In addition to these endocrine effects, perivascular and epicardial adipose tissue can exert direct effects on the adjacent vascular wall or myocardium, respectively, through the paracrine release of bioactive mediators. Additionally, adipose factors can reach the lumen of the adjacent vessel and travel downstream, which regulates the biology of the entire vascular beds in a “vasocrine” manner [18]. 

Vascular tone, inflammation, vascular smooth muscle cell migration, endothelial function, and vascular redox state are all under the regulation of adipose tissue-derived products, including adipokines [18]. The adipose tissue regulates the vascular tone in healthy conditions through the release of molecules with vasorelaxant properties, such as adiponectin [29], hydrogen sulfide [30], and palmitic acid methyl ester [31]. The decreased production of all these mediators in obesity and insulin resistance may contribute to the obesity-related vasomotor dysfunction [32]. Adipose tissue, mainly when located at the perivascular site, modifies the local production of angiotensinogen, which is crucial for the circadian regulation of blood pressure [33]. NADPH oxidase overactivity is a potent source of superoxide radicals that induce a shift toward the obesity-related pro-oxidative state, and enhances the cardiovascular risk factor-dependent organ damage [34]. The activity of certain isoforms of NADPH oxidase is inhibited by anti-oxidant adipokines such as adiponectin and omentin-1 [29,35] but promoted by pro-oxidative adipokines such as leptin, resistin, and subsequent insulin resistance, which contributes to a pro-oxidative state, endothelial dysfunction, and accelerated biological senescence [36,37,38], with the adiponectin to leptin ratio being a promising index to estimate the obesity-associated cardiometabolic risk [39,40]. Vascular inflammation is further promoted by the expression of endothelial cell adhesion molecules, which are induced by adipokines such as interleukin 32 (IL-32), visfatin, IL-1β, and the tumor necrosis factor (TNF) [41,42,43]. Certain adipokines, such as osteopontin and leptin (known as ‘adipofibrokines’), also promote cardiac fibrosis by inducing the synthesis of the extracellular matrix in cardiac fibroblasts [44].

## 3. Specific Adipokines

**Figure 1:**
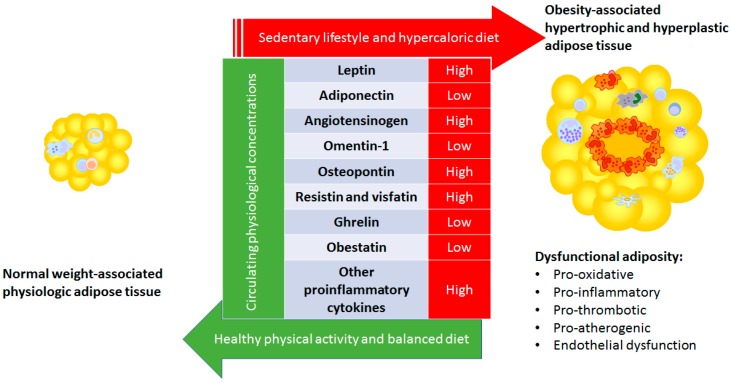
Main changes observed in the dysfunctional adipose tissue-derived adipokines.

Among the huge variety of adipokines produced by adipose tissue, acute-phase reactants (C-reactive protein, serum amyloid A, plasminogen activator inhibitor 1, haptoglobin), cytokines (TNF-α, IL-6, IL-10, IL-R1a, TGFβ), chemokines [MCP-1, macrophage inflammatory protein-2 (MIP-2), CCL2, CCL5, IL-8/CXCL8, IFNγ-inducible protein 10/ CXCL10], damage-associated molecular pattern molecules (DAMPs, tenascin C, calprotectin, heat shock protein 72, and HMGB1), as well as pro-inflammatory (leptin, resistin, osteopontin, chemerin, WNT5A, among others) and anti-inflammatory (adiponectin, SRFP5, omentin, ghrelin, and lipocalin-2) factors can be found [14]. Obesity is associated with an altered secretion of adipokines that, in fact, translates into increased cardiovascular risk in patients with an excess of dysfunctional adiposity. For the purpose of this review, we have focused on selecting the main adipokine and some of the emerging adipokines associated with augmented cardiovascular risk (Figure 1).

### 3.1. Leptin

Leptin, which is the obesity gene product, participates in the control of body weight by regulating food intake and energy expenditure [45] and, as a key hormone in energy homeostasis, also regulates neuroendocrine function, including reproduction. In the healthy state, leptin induces a balanced effect on the control of blood pressure by modulating the sympathetic activity-dependent vasoconstriction, and the endothelial release of nitric oxide, as well as the angiotensin II-dependent vasoconstriction [46,47]. The smooth muscle layer represents an important target for the vascular effects of leptin. This adipokine decreases passive wall tension and Ang II-induced vasoconstriction operating directly on vascular smooth muscle cells (VSMCs). Leptin inhibits the basal proliferation of aortic VSMCs and inhibits the Ang II-induced cell growth of VSMCs, in which leptin receptor activation is related to nitric oxide (NO)-dependent endothelial auto-regulatory capacity. Leptin induces endothelial-dependent vasodilation by activating a PI3-kinase-independent Akt-endothelial NOS (eNOS) phosphorylation pathway. This adipokine decreases passive wall tension and Ang II-induced vasoconstriction by up-regulating inducible Nitric-oxide sintase (iNOS) through mechanisms involving JAK2/STAT3 and PI3K/Akt pathways in VSMCs. Contradicting effects on different cell types and in different disease substrates have been described when evaluating leptin physiology and comparing health and disease states [48], since obesity induces an organ-specific leptin resistant state. Therefore, hyperleptinemia in obesity may arise as a compensatory mechanism to overcome leptin resistance. Despite higher circulating-levels, the previously mentioned vascular effects are attenuated. Whereas endothelial leptin signaling is considered to be protective against neointima formation in the healthy state, obesity-induced leptin resistance can reverse this balance toward an atherogenic phenotype.

Additionally, leptin resistance effects on the reproductive homeostasis entails hypogonadism that further aggravates the obese phenotype by changing the body composition, and easing hyperglycemia, hyperinsulinemia, and insulin resistance with its associated excess of cardiovascular risk [49]. 

In the clinical setting, and despite the previously mentioned physiology, the relevance of hyperleptinemia as an isolated cardiovascular risk marker or mediator seems to be small [45] or even absent [50] due to available prospective studies. Obesity is a complex disease that affects a wide range of pathogenic mechanisms in which only the holistic management of the disease has proven to have relevant efficacy. Consistently, of note, leptin decreases and its physiology is restored after bariatric surgery [51], which is in line with the improvement of the cardiovascular risk-factor milieu described post-surgically [52].

### 3.2. Adiponectin

Adiponectin is an adipokine expressed almost exclusively in adipose tissue. It can be found in plasma in three major oligomeric forms, which include a low-molecular-weight, middle-molecular-weight, or high-molecular-weight adiponectin. Adiponectin increases insulin sensitivity and exerts anti-inflammatory actions [53]. Plasma adiponectin concentrations decreased in patients with obesity, and high-molecular-weight adiponectin better predicts insulin resistance and the metabolic syndrome in humans [54]. In the clinical practice, adiponectin levels also correlate positively with coronary heart disease recurrence and all-cause and cardiovascular mortality among patients with established cardiovascular disease [55] as well as in elevated circulating total adiponectin levels, which were associated with lower 10-year CVD risk in adults without previous CVD, independently of other established CVD risk factors.

Moreover, the adiponectin/leptin ratio has been suggested as a maker of adipose tissue dysfunction and correlates with insulin resistance more closely than adiponectin or leptin alone or even the HOMA index, which is a surrogate of insulin resistance [39]. Reportedly, this ratio decreases with an increasing number of metabolic risk factors reflecting the functionality of adipose tissue, which have been proposed as a predictive marker for the metabolic syndrome [56]. Bariatric surgery induced weight-loss and induced sustained increases in adiponectin plasmatic levels [57] in parallel with the whole improvement of the metabolic profile, and the decrease in leptin levels observed post-surgically.

### 3.3. Angiotensinogen

Angiotensinogen is the main precursor of the renin–angiotensin system, which is a pivotal mechanism for the regulation of blood pressure and homeostasis of water and sodium through actions of angiotensin II. In addition to hepatic angiotensinogen production, adipose tissue has also shown to be a quantitatively important source of angiotensinogen [58]. The 10 N-terminal amino acids of angiotensinogen are cleaved by renin to provide angiotensin I, which is further transformed into angiotensin II by the angiotensin converting enzyme (ACE) [59]. Angiotensin II causes vasoconstriction, increased blood pressure, and release of aldosterone from the adrenal cortex in the vascular-tone axis [59,60] and also induces the differentiation from preadipocytes to mature adypocytes [58]. The expression of angiotensinogen is enhanced in the setting of obesity and insulin resistance and exerts its pro-oxidative, pro-inflammatory, and pro-fibrotic functions via NF-κB signaling enhancement and NADPH oxidase activity induction. Therefore, obesity-induced angiotensin excess is a cornerstone in the management of obesity-associated hypertensive disease [59]. Bariatric surgery is associated with a 70% rate of resolution of the hypertensive disease, since it improves all the mechanisms that link obesity with hypertension, including the renin-angiotensin-aldosterone system (RAAS) overactivity [61] along with sympathetic activity-dependent vasoconstriction and insulin resistance.

### 3.4. Omentin-1

Omentin is encoded by two genes, known as omentin-1 and omentin-2, with the former being the major circulating form. Omentin-1 is mainly expressed in the stromal vascular cells of visceral adipose tissue that plays an anti-inflammatory, anti-oxidative, and insulin-sensitizer role [62]. In obesity, omentin-1 levels are decreased and are inversely correlated to BMI, waist circumference, and markers of metabolic syndrome. Omentin-1 is also decreased in patients with coronary heart disease. During diet-induced weight loss, omentin-1 levels tend to increase over time, which is an observation that further strengthens the link between omentin and obesity [63].

In vitro, omentin-1 enhances insulin-stimulated glucose uptake in human adipocytes by activating Akt signaling pathways. Furthermore, circulating omentin-1 concentrations are associated with endothelial dysfunction in patients with impaired glucose tolerance by inhibiting the NADPH oxidase activity and enhancing the TNF-induced vascular cell adhesion molecule 1 (VCAM1) expression [64,65,66].

Bariatric surgery induces variable behavior in omentin-1 levels. Most patients exhibit an increase in the immediate postoperative period, even before induction of weight loss, and these levels were kept high up to one-year post-bariatric intervention [67]. However, not all patients demonstrated this omentin-1 increase following bariatric surgery. In fact, about 20% saw their levels decrease, but still demonstrated a resolution of their diabetic status over a short-term post-surgery [67]. The impact of this observation still needs further evaluation. 

### 3.5. Osteopontin

Osteopontin is a 40-kDa to 80-kDa secreted matrix glycoprotein [68]. In adipose tissues, osteopontin is expressed by adipocytes and by the stromal-vascular cells (lymphocytes, endothelial cells, macrophages, vascular smooth muscle cells, and mesenchymal stem cells). Osteopontin expression in human macrophages is also upregulated by a variety of pro-inflammatory mediators, including TNF-α, IL-6, and oxidized LDL, known to be elevated in obesity [68,69,70], type 2 diabetes, and cardiovascular disease [71]. Osteopontin acts as a pro-inflammatory cytokine that has been characterized as a major component of cell-mediated immunity [72], acting as an important attachment and signaling molecule, with the main ability to interact with integrin surface receptors [73]. Although the mechanisms of osteopontin related to cardiovascular diseases are not clear, different possible mechanisms exist. One of these possible mechanisms is osteopontin increasing the risk of atherosclerosis by increasing endothelial cell migration via αvβ3 ligand. Other possible mechanisms include the macrophage activation that entails calcifications and inflammatory processes associated with coronary artery disease [74]. Osteopontin is also expressed and released into the circulation from Kupffer cells, macrophages, stellate cells, and hepatocytes in non-alcoholic fatty-liver disease. Thereby, osteopontin may also contribute to the increased risk of cardiometabolic diseases observed in non-alcoholic fatty-liver disease [75]. Consistently, excessive osteopontin is associated with increased left ventricular stiffness and systolic dysfunction in patients with hypertensive heart disease and heart failure [76]. Osteopontin is also highly expressed in human atherosclerotic plaques [77].

Diet-induced weight loss is associated with a reduction in plasmatic and gene expression levels of osteopontin in visceral fat in obese animals and humans [78], which reflects an improvement in systemic and local inflammation. Although diet-induced weight loss is paralleled by a decrease of circulating osteopontin levels, a substantial increase in circulating osteopontin concentrations despite downregulation of local osteopontin expression in white adipose tissue in obese patients after bariatric surgery. Further investigations are needed to differentiate whether these changes are secondary to alterations of bone metabolism or an adaption to weight loss [71]. 

### 3.6. RESISTIN and VISFATIN

Resistin is a polypeptide that is secreted by adipose-tissue resident-macrophages [79]. Resistin concentrations are increased in obesity, due to its circulating levels being involved in the pathophysiology of inflammation-induced insulin resistance in humans. This association has been confirmed by prospective case-control studies that have found an increased risk of developing type 2 diabetes in subjects with elevated baseline levels of resistin. Some reports have also correlated resistin with hypertension, with a mechanism that is not fully understood, with atherogenic dyslipidemia, by modulating SREBP1-SREBP2 pathways, and with proprotein convertase subtilisin/kexin type 9 (PCSK9). Human resistin also plays a major regulatory role in the inflammatory response. Resistin upregulates the expression of pro-inflammatory cytokines such as TNF-α, IL-6, IL-12, and monocyte chemoattractant protein (MCP)-1 in monocytes, macrophages, and hepatic stellate cells via the nuclear factor-κB (NF-κB) pathway. It might also play a significant role in the modulation of interactions between endothelial cells and monocytes/macrophages, in the pathogenesis and progression of atherosclerosis. Resistin induces the expression of endothelin-1 in endothelial cells, which contributes to endothelial dysfunction. Resistin increases the expression of the cellular adhesion molecule VCAM-1 and the MCP-1, which are the factors in the formation of early atherosclerotic lesion, and induces the proliferation of smooth muscle cells. This suggests the action of these hormones is restenosis of coronary lesions in patients with diabetes [62]. Consistently, high resistin levels also correlate with increased risk of all-cause and cardiovascular death [80]. It is also remarkable that the excess of resistin is among the cytokines that define the metabolic unhealthy state, associated with obesity, as demonstrated by the decrease of circulating resistin levels after bariatric surgery [81,82]. 

Visfatin is an adipocytokine secreted by adipocytes, macrophages, and inflamed endothelial tissue that is elevated in obesity, insulin resistance, and type 2 diabetes mellitus. Visfatin acts as a pro-inflammatory mediator, with the ability to induce matrix metalloproteinase (MMP)-9 and NF-κB in monocytes and in human endothelial cells, which plays an important role in the pathogenesis of vascular inflammation in obesity and type 2 diabetes, and leads to atherosclerotic plaque instability. High visfatin levels also correlate with an increased risk of adverse cardiac events among patients with myocardial infarction [41,83,84]. It is tempting to hypothesize that this adipokine could contribute to the inflammatory state and increased risk for cardiovascular events characterizing patients with type 2 diabetes in a direct way rather than with diabetes itself. Plasma levels of visfatin are gradually reduced after bariatric surgery-related weight loss [82].

### 3.7. Ghrelin

Ghrelin was first described as the endogenous ligand for the growth hormone secretagogue receptor, which is the classic function described for this peptide [85]. Despite this, during the last two decades, the research of ghrelin functions has mainly focused on appetite, adiposity, and metabolism. Circulating ghrelin can be found in two main isoforms (namely acylated and deacylated-ghrelin) that exhibit antagonist effects on hepatic-glucose output, to the point that some authors have even suggested considering them as separate hormones [86]. Growing evidence supports the important role of ghrelin in the control of cardiovascular homeostasis through central and peripheral mechanisms. There are multiple mechanisms underlying the effects of ghrelin on the CVD, mediated through both direct and indirect physiological actions, including increased Growth hormone (GH) levels, improved energy balance and direct actions on CV cells, regulation of autonomic nervous system activity [87], and modulation of autophagy [88], which is an important catabolic process involved in multiple physiological processes. Ghrelin is able to block the renin-angiotensin system improving hypertension and cardiovascular disorders. Chronic treatment with ghrelin decreases mean arterial pressure and systemic vascular resistance in chronic heart failure without increasing heart rate because its administration decreased plasma levels of norepinephrine. Meanwhile epinephrine levels were increased [89,90,91]. 

As previously mentioned, major circulating isoforms of this hormone are the acylated and the desacyl ghrelin. The acylation of ghrelin in the endoplasmic reticulum is catalyzed by the ghrelin O-acyltransferase, which is mainly modulated by nutrient availability, particularly by ingested medium-chain free fatty acids. The opposite effects of ghrelin isoforms on hepatic glucose metabolism have been reported with desacyl ghrelin suppressing glucose release by hepatocytes as well as antagonizing the acylated ghrelin-induced increase in hepatic glucose output In vitro [92]. Under pathological conditions, such as obesity, the liver responds to the massive influx of lipids from the blood by upregulating the biogenesis of lipid droplets, as a mechanism of defense against free fatty-acids (FFA) toxicity. An impaired autophagic flux increases triglycerides storage in lipid droplets, which leads to the development of non alcoholic fatty-liver disease (NAFLD). Acylated and, to a lesser extent, desacyl ghrelin activates autophagy in rat hepatocytes, which contributes to the amelioration of NAFLD [88].

On the other hand, cardiomyocytes rely critically on the housekeeping mechanisms of proteostasis, such as autophagy, due to their limited ability to divide. During myocardial ischemia, stimulation of autophagy by the pharmacological inhibition of the mTOR pathway protects against post-infarction remodeling. Nonetheless, the activation of cardiac autophagy requires a tight regulation with both deficient and excess autophagy being detrimental, which leads to proteotoxicity or excessive degradation of intracellular components, and, in turn, cardiomyocyte death, respectively. The myocardium constitutes a source of ghrelin and accumulating evidence has demonstrated the cardioprotective effects of ghrelin by increasing myocardial contractility, decreasing cardiac fibrosis, inflammation, and apoptosis of cardiomyocytes as well as protecting the myocardium against ischemia/reperfusion injury. During acute cardiac ischemia, both ghrelin isoforms, but in particular desacyl ghrelin, markedly reduced infarction size and preserved cardiac function, in part, by the activation of autophagy to remove dysfunctional mitochondria after myocardial infarction in mice. Intraperitoneal administration of desacyl ghrelin in obese, diabetic mice protects against diabetic cardiomyopathy via the pro-survival AMPK and ERK1/2 pathways. Moreover, chronic intraperitoneal administration of ghrelin improves autophagy in vascular smooth muscle cells from rats with vascular calcification in an AMPK-dependent manner. Taken together, ghrelin also minimizes hypoxia-induced cardiac injury and decreases vascular calcification by activating autophagy through AMPK-dependent mechanisms [88]. Furthermore, ghrelin administration could protect against ischemia and reperfusion injury, attenuate post-infarction ventricular dysfunction and remodeling, and improve the prognosis of myocardial infarction and heart failure [90,91,93,94,95,96]. 

Total ghrelin levels decrease after eating and in the context of hypercaloric diet, but bariatric surgery does not substantially modify them. Gastrectomy specifically reduces the desacyl ghrelin levels, due to the resection of the gastric fundus, which is the major production site of the hormone. This effect entails an increase in the acylated/desacyl ghrelin ratio. The increase in acylated ghrelin levels after gastrectomy is involved in the induction of liver autophagy, and entails a beneficial effect on the liver function mediated, among others, via a decrease in lipogenesis as well as an increase in autophagy and mitochondrial β-oxidation. The decrease in desacyl ghrelin after sleeve gastrectomy contributes to the reduction of lipogenesis, whereas the increased acylated ghrelin levels stimulate AMPK-activated mitochondrial FFA β-oxidation and autophagy. These results support the notion that both ghrelin isoforms constitute key elements involved in the improvement of NAFLD after bariatric surgery. 

### 3.8. Obestatin

Obestatin is a gastrointestinal peptide derived from preproghrelin, mainly produced throughout the gastrointestinal tract but also in the pancreas, testes, and brain [97]. Decreased plasmatic obestatin levels have been documented in patients with excess adiposity, impaired glucose control, metabolic syndrome, insulin resistance, and diabetes mellitus. Obestatin exerts its beneficial actions on both metabolism and cardiovascular function It has been reported to decrease food intake and body weight, to promote β-cell mass, and appears to be involved in blood pressure regulation and exerts beneficial effects on endothelial function [97]. Obestatin modulates endothelial function directly, in an NO-dependent manner [98] and, indirectly, via anti-inflammatory effects on human endothelial cells, by decreasing TNF-α-induced VCAM-1 [99]. The biological actions of obestatin in the modulation of the endothelial function suggests that it might play a role in the regulation of blood pressure, mainly in the setting of obesity and its complications. In addition to its vascular effects, obestatin has been reported to confer dose-dependent protection reducing the infarct size and the contractile dysfunction in an animal model of the ischemia–reperfusion myocardial injury [100]. Changes in the physiology of obestatin following bariatric surgery still needs further evaluation. 

### 3.9. Other Pro-Inflammatory Cytokines

IL-32, which is also termed as a TNF-α-inducing factor, is a recently described cytokine produced by immune and non-immune cells, which exerts important roles in the pathogenesis of infectious, autoimmune, and inflammatory diseases. Increased IL-32 expression has been found in visceral adipose tissue from patients with obesity promoting inflammation and extracellular matrix remodeling and contributing to the development of obesity-associated comorbidities. Increased circulating levels of IL32 in human obesity and obesity-associated diabetes decrease after weight loss and are also closely associated with inflammatory cytokines such as IL1β, interferon (IFN)γ, or TNFα, which induce the expression of IL32 and, in turn, IL32 also stimulates IL8, IL6, IL1β, and TNFα production. This constitutes a classical pro-inflammatory mediator with relevant functions in angiogenesis, extracellular matrix remodeling, and apoptosis [101].

## 4. Conclusions 

A key factor underlying the etiopathogenesis of obesity is the dysfunctional adipose tissue, characterized by adipocyte hypertrophy, exacerbated inflammation, increased fibrosis, and impaired vascular function and structure. Adipose tissue influences and communicates with many other organs by releasing pro-inflammatory and anti-inflammatory bioactive molecules, known as adipokines. Altered expression of these molecules plays critical roles in impairing whole-body homeostasis contributing to the initiation and progression of obesity-induced metabolic complications. While this review has focused on some of the main adipokines, the influence of many other factors like serum amyloid A [102], caveolin-1 [103], fibroblast growth factors [104], tenascin, and calprotectin [105] among others should not be disregarded. Moreover, the intense cross-talk between adipose tissue and other metabolically active organs like skeletal muscle, myocardium, and vascular smooth muscle cells via myokines needs to be contemplated [28,106,107].

Considering the expected increase in the prevalence of obesity in the coming decades, a more detailed study of the functions and mechanisms of the pleiad of adipokines will permit a better understanding of the pathogenesis of obesity-linked disorders as well as more focused therapies for the management of obesity and its complications. Thinking beyond the metabolic functions of adipose tissue is needed given the wide-ranging and versatile effects of the multiple adipokines and their plentiful impact on diverse disease areas. 

## 5. Review Criteria

A search for original articles and reviews published focusing on the pathophysiology of obesity-associated excess of cardiovascular risk was performed in PubMed and MEDLINE using the following search terms (or combination of terms): “obesity,” “adiposity,” “fat mass,” “epicardial fat,” “visceral fat,” “dysfunctional fat,” “comorbidity or comorbidities,” “outcome,” “mortality,” “adipokines,” “cardiovascular disease,” “coronary heart disease,” “stroke,” “myocardial infarction,” “body composition,” and “miokines.” Only English-language, full-text articles were included. Additional articles that were identified from the bibliographies of the retrieved articles were also used.
